# A Cross-Sectional Study Investigating Associations between Personality Traits, Glycemic Control, and BMI in Persons with Diabetes: Lolland-Falster Health Study, Denmark

**DOI:** 10.3390/ijerph21091231

**Published:** 2024-09-18

**Authors:** Zandra Overgaard Pedersen, Bettina Ewers, Cathrine Lawaetz Wimmelmann, Allan Kofoed-Enevoldsen, Rasmus Køster-Rasmussen, Christian Couppé, Erik Simonsen, Jesper Dammeyer

**Affiliations:** 1 Steno Diabetes Center Copenhagen, Department of Diabetes Care, 2730 Herlev, Denmark; 2 Neuromuscular Center, Department of Neurology, Copenhagen University Hospital, Rigshospitalet, 2100 Copenhagen, Denmark; 3 Unit of Medical Psychology, Department of Public Health, University of Copenhagen, 1353 Copenhagen, Denmark; 4 Centre for Childhood Health, 2300 Copenhagen, Denmark; 5 Steno Diabetes Center Zealand, Department of Endocrinology, Nykøbing Falster Hospital, 4800 Nykøbing Falster, Denmark; 6 The Research Unit for General Practice, Department of Public Health, University of Copenhagen, 1353 Copenhagen, Denmark; 7 Department of Physical and Occupational Therapy, Bispebjerg and Frederiksberg University Hospital, 2400 Copenhagen, Denmark; 8 Institute of Sports Medicine Copenhagen, Department of Orthopedic Surgery, Bispebjerg and Frederiksberg University Hospital, 2400 Copenhagen, Denmark; 9 Center for Healthy Aging, University of Copenhagen, 2200 Copenhagen, Denmark; 10 Department of Clinical Medicine, University of Copenhagen, 2200 Copenhagen, Denmark; 11 Mental Health Services East, Copenhagen University Hospital, 4000 Roskilde, Denmark; 12 Department of Psychology, University of Copenhagen, 1350 Copenhagen, Denmark

**Keywords:** Big Five personality model, diabetes, personality traits, glycemic control, BMI, HbA1c

## Abstract

There is a growing focus on person-centered care, emphasizing the importance of respecting inter-individual differences and implementing individualized treatment initiatives. Prior research has established an association between personality traits, body mass index, and health-related behaviors. The aim of this study was to explore the potential of personality trait assessments in identifying individuals at risk of glycemic dysregulation and increasing BMI. This cross-sectional study used a dataset comprising 140 participants with diabetes who completed the Big Five personality trait questionnaire from the Lolland-Falster Health Study. Logistic regression was used to investigate associations between personality traits, glycemic control, and BMI ≥ 25. No significant associations between personality traits and glycemic control were found. There was a significant association between agreeableness and lower odds of BMI ≥ 25 in the unadjusted analysis (OR 0.54 (0.34–0.86)), which persisted after adjusting for sex, age, and education (OR 0.54 (0.33–0.89)). No significant association between glycemic control and personality traits was observed in this small sample study. However, higher levels of agreeableness were associated with a lower likelihood of having a BMI of ≥25. This preliminary study suggests that integrating personality assessments could help identify individuals at risk of increasing BMI. These findings highlight the potential of using personality traits to guide targeted interventions, offering a direction for future research.

## 1. Introduction

The global increase in diabetes is influenced by factors such as genetics, aging, lifestyle, and increasing body mass index (BMI) in the population [1,2,3]. In Denmark, rural areas such as Lolland-Falster have a high prevalence of diabetes, and the prevalence is associated with elevated HbA1c levels and higher BMI, which contributes to insulin resistance and impaired glycemic control [4,5,6,7]. Despite massive advancements in antidiabetics over the past decade, achieving desirable HbA1c levels remains challenging [4]. While adherence to antidiabetic medications is the cornerstone of present-day diabetes treatment, adherence to diet and physical activity interventions is essential in diabetes management due to the documented effects on HbA1c and BMI [8,9,10,11,12,13,14,15,16,17] as well as the health benefits beyond HbA1c reduction and weight loss [18,19]. Still, several studies have documented that adherence to these treatment initiatives can be challenging [17,20,21,22]. This emphasizes the need to explore methods to improve adherence, identify individuals with the greatest needs, and provide personalized guidance on lifestyle recommendations. There is a growing focus on a person-centered approach within diabetes management, which encompasses medication, social determinants, and therapist guidance to enhance adherence [23]. This approach aims to efficiently support individuals in achieving success with adherence to lifestyle interventions and facilitate the attainment of desirable glycemic control mediated by lifestyle modifications [23]. Part of delivering person-centered treatment is to respect the importance of different behaviors and personality characteristics [24]. By accepting individualized needs and differences in behavior, the healthcare professional can individualize the treatment and guidance to the individual’s needs and potentially explain and predict clinical outcomes. One determinant that can be important for receiving information, as well as adherence, is personality traits [25,26]. A personality trait is defined as thoughts, emotions, and behaviors that differentiate individuals from each other [27,28]. These traits should not be situation-dependent and must exhibit consistency across various situations and contexts in which the individual is involved [29]. The Big Five trait theory, upon which this study is based, consists of the following traits: openness to experience, conscientiousness, extroversion, agreeableness, and neuroticism [30]. By identifying personality traits that are particularly predisposed to dysregulation and increasing BMI, we can potentially determine which traits need clinical attention in relation to lifestyle interventions. This information might enable healthcare professionals to allocate resources more efficiently and develop interventions specifically tailored to high-risk groups. Specifically, a personalized approach has the potential to enhance adherence to tailored dietary and physical activity interventions. Individuals with certain personality traits may benefit more from alternative strategies than from standard one-on-one patient–healthcare provider interactions. Hence, this population-based study aims to explore the potential of personality trait assessments in identifying individuals at risk of glycemic dysregulation and increasing BMI. By investigating the relationships between personality traits, glycemic control, and BMI in individuals with diabetes, this research aims to provide insights that future studies can build upon to develop and investigate tailored interventions.

## 2. Materials and Methods

### 2.1. Study Design and Population

#### 2.1.1. Study Population

This cross-sectional population study is based on data from the Danish Lolland-Falster Health Study (LOFUS). LOFUS is a prospective cohort study that assembles data on health, lifestyle, and socioeconomic determinants [31]. The protocol article and questionnaire development have been published elsewhere [31,32]. Individuals aged ≥18 years were randomly selected from the Danish Civil Registration System and invited to participate in LOFUS [31]. The health examination consisted of a web-based questionnaire and physiologic examinations in one of three satellite units [31]. The participation rate in LOFUS was 34% [33]. The LOFUS database includes responses from 18,949 citizens [34]. The Big Five Inventory (BFI-10) questionnaire was included in a survey to a subsample of the participants ≥60 years of age. Participants who answered the supplemental BFI-10 survey and had diabetes (N = 140) were included in this study. LOFUS was approved by the Scientific Ethics Committee for Region Zealand (journal number: SJ-421). The present study was approved by the General Data Protection Regulation (P-2023-69). Data were accessed for research purposes on 17 March 2023.

#### 2.1.2. Diabetes Definition

Diabetes was defined as a self-reported diabetes diagnosis from the questionnaire or, if the participant declared that they did not have a known diabetes diagnosis, a clinical presentation of HbA1c ≥ 48 mmol/mol during the health examination. Hence, the following answers were extracted: “Do you have a known diabetes diagnosis? (Yes/No)”. Clinical data for HbA1c were extracted from the database. LOFUS did not distinguish between type 1 diabetes (T1D) and type 2 diabetes (T2D). Thus, participants in this study were managed as one homogeneous group and were a representative sample of individuals with T1D or T2D from the background population in Lolland-Falster from 2016 to 2020.

### 2.2. Independent Variables

#### Personality Traits

Personality traits were measured by the use of a previous Danish translation of the BFI-10 questionnaire [35]. The BFI-10 questionnaire consists of 10 items, each rated on a five-point Likert scale ranging from “strongly disagree” to “strongly agree” [36]. The scale comprises two items for each of the five personality traits of the Five Factor Model: neuroticism, extroversion, openness to experience, agreeableness, and conscientiousness [37]. Thus, each personality trait was analyzed on a scale ranging from 2 to 10. The BFI-10 questionnaire is reliable and valid, concordant with the Revised Neo Personality Inventory (NEO-PI-R) [38].

### 2.3. Outcome Variables

#### 2.3.1. HbA1c

At the LOFUS health examination, 12 mL of blood was collected using BD vacutainers from all participants >15 years of age. Non-fasting HbA1c samples were collected and preserved at 21° until the blood samples were analyzed at the clinical biochemical department at Nykøbing Falster Hospital. HbA1c blood samples were estimated by high-pressure liquid chromatography with a Tosoh 723G11.

#### 2.3.2. Glycemic Control

The American Diabetes Association (ADA) recommends an HbA1c level of ≤53 mmol/mol for non-pregnant adults without significant hypoglycemic events [39]. The Danish Endocrinological Society (DES) suggests that HbA1c upon disease onset should be 48 mmol/mol and 53 mmol/mol in T2D and T1D, respectively [40,41]. Studies have implied that the risk of microvascular complications decreases if an HbA1c level of ≤53 mmol/mol is achieved early in the disease course [39,42]. Our study population was thus divided into two categories depending on the degree of glycemic control: (1) well-regulated glycemic control (38–53 mmol/mol) and (2) dysregulated glycemic control (≥54 mmol/mol).

#### 2.3.3. BMI

BMI was extracted from bioimpedance measurements performed during the health examination. BMI is calculated by weight in kilograms divided by height in meters squared (kg/m^2^). The World Health Organization (WHO) classifications for normal weight (BMI < 25) and overweight (BMI ≥ 25) [43] were used to divide the study population into two groups: BMI < 25 and BMI ≥ 25.

### 2.4. Statistics

Descriptive statistics of participant characteristics were performed for the whole sample, and participants were divided into the two glycemic control sub-groups. Odds ratios (ORs) and confidence intervals (CIs) were determined to investigate the association between personality traits and dysregulated glycemic control using unadjusted and adjusted bivariate logistic regression. Nominal HbA1c measures were used as indicators of glycemic control and outcome variables. Unadjusted and adjusted bivariate logistic regression models were performed to investigate the associations between personality traits and BMI ≥ 25. The adjusted bivariate logistic regression models for glycemic control and BMI comprised adjustments for sex, age, and education. Additionally, the glycemic control models were adjusted for antidiabetic medications and BMI, given that increasing BMI can potentially dysregulate glycemic control. All statistical analyses were applied with a 5% level of significance and were managed with SPSS version 28.0.1.0.

## 3. Results

### Main Results

The characteristics of participants with diabetes who completed the BFI-10 questionnaire are presented in Table 1. The OR for the association between the Big Five personality traits and dysregulated glycemic control is presented in Table 2. No significant association was found between personality traits and the OR for dysregulated glycemic control (HbA1c ≥ 54 mmol/mol). In Table 3, the association between personality traits and BMI ≥ 25 is presented. Agreeableness was associated with reduced odds of having a BMI of ≥25 (OR 0.54 (0.34–0.86)). This association remained after adjusting for sex, age, and education (OR 0.54 (0.33–0.89)).

## 4. Discussion

### 4.1. Main Findings

The present study found an association between Agreeableness and reduced odds of having a BMI of ≥25. This association persisted after adjusting for sex, age, and education. No associations were observed between personality traits and glycemic control.

### 4.2. Other Studies

Studies investigating the association between personality traits and glycemic control are limited. Some studies have investigated the associations between personality traits, self-care, adherence, BMI, and glycemic control in individuals with diabetes [44,45,46,47,48,49,50]. In the existing literature, the Big Five personality traits were only applied in five studies, and they had substantial variation in the examined outcomes, encompassing self-care, adherence, and glycemic control [45,46,47,48,50].

Neuroticism is known for being associated with the experience of stress, sensitivity, and the risk of mental and physical disorders [51]. Previous studies have revealed that neuroticism is associated with both increased and reduced HbA1c levels in patients with T2D [46,47]. One of these studies found that the odds of dysregulated glycemic control (HbA1c ≥ 54 mmol/mol) were three times higher (OR 3.19) for participants with high neuroticism scores in a sample consisting of 214 participants [46]. However, the study used a different instrument to evaluate personality traits [46], which measured only four personality dimensions, as openness and extroversion were combined into one trait [46]. Our study did not find any association between Neuroticism and glycemic control or BMI ≥ 25. The observed association between Neuroticism and reduced odds of dysregulated glycemic control in previous studies may have been due to anxious blood glucose monitoring with a strong response and, in turn, the aggressive use of bolus insulin, which may have led to a high prevalence of hypoglycemia, resulting in well-regulated HbA1c. However, an HbA1c level within the target range is not considered good glycemic control when it is due to large fluctuations in blood glucose variability. One meta-analysis investigated the associations between personality traits and HbA1c in six samples (n > 26.000) in which individuals with and without diabetes were included [45]. This study found that higher levels of conscientiousness were associated with reduced odds of HbA1c levels above 48 mmol/mol (OR 0.85) [45]. The findings from our study did not reveal any significant association between conscientiousness and dysregulated glycemic control. This can probably be attributed to the small sample size, which increases the risk of type II error [52]. The determination of when HbA1c levels are considered dysregulated appears to have significance in identifying which personality traits are correlated with dysregulation. Hence, a more nuanced classification of dysregulation could prove beneficial for exploration in future research.

### 4.3. Strengths and Limitations

The most significant limitation of this study is the small sample size. The small sample size limits the generalizability of our findings and may only provide a preliminary understanding of the relationships between personality traits, BMI, and glycemic control.

#### 4.3.1. Psychometrics

The BFI-10 questionnaire only measures the five broad personality domains [36]. Instead of analyzing the five domains, detailed facet-level analyses could be performed. One study found that facet N5, impulsiveness, was associated with weight gain corresponding to 10% of the individual’s body weight after weight loss [53]. By addressing facets instead of domains, it may be possible to identify specific facets related to BMI and dysregulated glycemic control. However, facet analyses require an instrument with significantly more items, such as the NEO-PI-R [54], which was not available in our study.

#### 4.3.2. Participant BMI

The average BMI for the total sample was 30.9 kg/m^2^, with a standard deviation of 5.5. A BMI equivalent to normal weight (BMI 18.5–24.9) [55] was thus represented in this study to a minor extent. A different approach could be to divide the population according to WHO’s obesity classes, in which the population is divided into the following three categorizations for BMI: obesity class I (30.0–34.9), obesity class II (35.0–39.9), and obesity class III (≥40) [55]. It is plausible that this division could mediate the differing results.

#### 4.3.3. Non-Response Bias

There is a risk of non-response bias if the characteristics of non-participants are systematically different from those of participants, which may affect the generalizability of the study [56]. In observational studies, respondents are frequently characterized by a high educational status and generally preferable health behavior [56]. Only 34% of the invited individuals participated in LOFUS, and they were characterized by a high educational level, income, and employment status [33]. A low level of these socioeconomic determinants is associated with BMI ≥ 25 and unhealthy lifestyles [57]. Hence, there is a risk that individuals characterized by unhealthy lifestyles, which can lead to increased BMI and dysregulated glycemic control, did not participate in the study due to non-response bias.

#### 4.3.4. Socioeconomic Determinants

Socioeconomic determinants are associated with an unhealthy lifestyle and an increased risk of developing T2D [58,59]. Furthermore, there is an association between a short education, physical inactivity, unhealthy dietary habits, and high BMI [58,60,61,62], all of which are factors that can have an impact on glycemic control. The personality traits openness and conscientiousness are associated with high education attainment [63]. Hence, we performed adjustments for educational level, which did not reveal any notable differences in the associations. However, it is plausible that adjustments for additional socioeconomic determinants could have impacted the results.

#### 4.3.5. Glycemic Control

HbA1c was used as a parameter to estimate glycemic control and was used to divide the participants into two categories: well-regulated glycemic control (HbA1c ≤ 53 mmol/mol) and dysregulated glycemic control (HbA1c ≥ 54 mmol/mol). These categorizations are based on the ADA’s recommendations for HbA1c in individuals with T1D without complications [39,40]. However, in practice, the target for HbA1c is individualized and depends on the individual’s comorbidities, age, and everyday challenges. Hence, it is recommended that elderly individuals with comorbidities and/or cognitive impairment have a less conservative target for glycemic control, and a target range of 64–69 mmol/mol is considered acceptable [64]. The average age of participants in this study was 66 years (Table 1). Since the incidence of comorbidities increases with advancing age and a less conservative approach to HbA1c is applied for these individuals, it is possible that HbA1c was at a level within the target range stipulated by the clinicians. The definitions of glycemic control used in our study may differ from the ones used in the clinic. Furthermore, individuals with T2D are considered well-regulated with an HbA1c of ≤48 mmol/mol. We were not able to distinguish between T1D and T2D; therefore, some of the individuals in the well-regulated group (≤53 mmol/mol) may not be within their target area. Thus, the reliability of the results concerning glycemic control in this cross-sectional study can be questioned, as there can be incongruence regarding when glycemic control is considered dysregulated.

#### 4.3.6. Cross-Sectional Study Design

One twin study investigated to what extent personality is inherited or learned [65]. This study estimated that heredity explains 39–58% of the variance in personality [65]. Traditionally, personality is perceived as a stable and consistent characteristic, which, to a minor extent, changes throughout life [66,67,68]. Studies have shown a general “maturation” of personality traits with age, which involves higher agreeableness and lower neuroticism scores [69]. However, personality traits are generally stable from the age of 30, but they can, to some degree, change due to life experiences [27,70,71]. The cross-sectional design of this study could thus be a limitation since the personality trait scores and HbA1c were only one-point measures. Follow-up measures of the personality trait scores or HbA1c levels could thus be incongruent with the present measures. However, the average age of the studied diabetes population was 66 years, at which personality traits are assumed to be relatively stable [67,68,71]. Therefore, it is plausible to assume that the personality traits of the respondents included in this study were relatively stable.

#### 4.3.7. Weight Stigma and Personality Traits

It is unclear whether there is an interaction between weight stigma and personality traits. There is an increasing focus on the impact of weight stigma and health-related outcomes [72]. Some of these associations may be mediated by personality traits. Studies have pointed out that weight stigma is related to unhealthy eating behavior, binge eating, and decreased motivation for diet and exercise interventions [73,74]. This fact complicates traditional weight strategies, in which the focus is on weight loss strategies, which often only have short-term results and can facilitate the emotion of failure in the individual. Beyond this, studies have shown a negative relationship between weight stigma and mental health, such as depression and anxiety [75], and a high degree of serological stress biomarkers, which may be related to the social construct of BMI [76]. All these factors may be associated with personality traits. Therefore, it is possible that the associations between personality traits and BMI are linked with the individual’s experience with weight stigma throughout life.

Furthermore, research has shown that being overweight is associated with lower self-esteem, negative self-image, and depressive symptoms in children and adolescents [77,78]. It has been highlighted that these factors can contribute to elevated Neuroticism scores [79], which are linked to undesirable health behaviors later in life. Additionally, increasing BMI may lead to social isolation due to social stigma, which can lead to lower extroversion scores. Increasing BMI may thus have a significant impact on personality development and health-related outcomes later in life. However, this association may be due to shared genetic risk factors; for example, major depressive events and higher Neuroticism scores share predisposed genetic risk factors, complicating the determination of causality [80,81].

#### 4.3.8. Other Individual Differences

Other individual differences were not investigated in this study. Self-efficacy is an individual’s expectation to succeed with a given task [82]. One study indicated that low self-efficacy can be a mediating factor for increasing BMI and inadequate adherence to antidiabetics [83]. Additionally, previous research has indicated that there is an association between a low self-efficacy score and dysregulated glycemic control and vice versa in individuals with diabetes [84,85]. It is thus plausible that a high degree of self-efficacy can be a decisive factor in achieving well-regulated glycemic control. Associations between the Big Five personality traits and health outcomes can be mediated by socio-cognitive factors [86,87]. One study investigated the impact of social contexts and personality traits on fruit intake [88]. The findings from this study revealed a positive association between conscientiousness and healthy food intake but found that the association was mediated by attitudes toward nutrition in the social environment [88]. Considering this assumption, it is possible that the association between agreeableness and BMI is mediated by attitudes in the social context.

#### 4.3.9. Perspective

Although our study did not reveal any significant associations with glycemic control, probably attributed to the risk of type II error, personalized guidance and lifestyle interventions that consider individual differences in personality traits seem relevant. Tailored interventions can be developed on the basis of these traits to facilitate optimal conditions for adherence. Individuals with high conscientiousness scores, characterized by responsibility and organization [89], may benefit from structured approaches, such as detailed meal planning, regular blood glucose monitoring, and goal setting. Conversely, individuals with high neuroticism scores, who are typically more anxious and stress-reactive [89] and may be prone to food cravings and binge eating, could benefit from stress management techniques. Individuals with high agreeableness scores are characterized as being emphatic and valuing deep interpersonal relationships [89]. Therefore, they may benefit from group settings centered around sharing experiences and peer learning. This setting could also be advantageous for individuals with high scores in extroversion who thrive in social interactions. Data regarding physical activity and diet were not available in our dataset; therefore, it was not possible to test the association or the mediating effect of these variables. Future research should investigate how personality traits influence adherence to physical activity and dietary interventions, aiming to identify the most effective intervention strategies for each trait. 

In our study, we were unable to distinguish between T1D and T2D, despite the unequivocal distinctions between these diseases. T1D is an autoimmune disease, which typically presents at a young age and requires insulin therapy from disease onset, whereas T2D usually develops later in life and can initially be managed with lifestyle modifications. The personality traits associated with glycemic control may vary between individuals with early-onset, insulin-dependent T1D and those with T2D. Future research could benefit from investigating these differences. The goal of present-day diabetes treatment is to prevent or delay the onset of late diabetic complications, with increasing attention to the impact of glycemic variability and fluctuations in the day-to-day blood glucose. Thus, future research should look beyond HbA1c levels and investigate which personality traits are associated with glycemic variability and the development of late diabetic complications.

## 5. Conclusions

This preliminary study suggests that integrating personality assessments could help identify individuals at risk of increasing BMI. Notably, high agreeableness was negatively associated with BMI, indicating a lower risk of BMI increasing. These findings highlight the potential of using personality traits to guide targeted interventions, offering a valuable direction for future research.

## Figures and Tables

**Table 1:** Characteristics of participants with diabetes who completed the BFI-10 questionnaire.

	**Total**	**Glycemic Control**	
		Well-regulated	Dysregulated
Total, n	140	80	60
Sex (Female/Male)	57 (40%)/84 (60%)	31 (39%)/49 (61%)	25 (42%)/35 (58%)
Age, years	66/9	66/9	67/9
Smoking,(Yes/No)	28 (20%)/112 (80%)	14 (17%)/66 (83%)	14 (24%)/45 (76%)
Education			
Public, or high school	26 (18.4%)	16 (20%)	9 (15%)
Suppl. Educational courses	11 (7.8%)	7 (8.8%)	4 (6.7%)
Vocational training	58 (41.1%)	26 (32.5%)	32 (53.3%)
2–3 y of further education	14 (9.9%)	7 (8.8%)	7 (11.7%)
2–4 y of further education	16 (11.4%)	12 (15%)	4 (6.7%)
>4 y of further education	4 (2.8%)	4 (5%)	0 (0%)
Other	12 (8.5%)	8 (10%)	4 (6.7%)
Personality traits			
Neuroticism *(n = 134, range 2–10)*	4.6/1.5	4.7/1.6	4.5/1.4
Extroversion *(n = 132, range 2–10)*	7.1/1.6	7.1/1.7	7.3/1.6
Openness *(n = 132, range 2–10)*	5.6/1.9	5.7/2.1	5.5/1.6
Agreeableness *(n = 132, range 2–10)*	7.4/1.3	7.5/1.4	7.4/1.4
Conscientiousness (*n = 134, range 2–10)*	7.7/1.4	7.8/1.3	7.6/1.6
BMI, kg/m^2^ *(n = 134)*	30.9/5.5	31.3/5.3	30.6/5.9
Height, m	171/8.9	171/8.7	171/9.2
Weight, kg	90.9/20.2	92/8.7	89.9/22
HbA1c, mmol/mol *(n = 140)*	54.2/11.6	46.4/4.2	64.6/10.2
Insulin (Yes/No)	37 (26.2%)/99 (70.2%)	9 (12%)/68 (88%)	21 (47%)/31 (53%)
Other antihyperglycemic agents (Yes/no)	99 (70.2%)/42 (29.8%)	59 (74%)/21 (26%)	40 (67%)/20 (33%)

Variables are specified as n/(%) or mean/standard deviation. Abbreviations: Suppl. = supplementary, y = years.

**Table 2:** Associations between personality traits and dysregulated glycemic control.

	Odds Ratios (ORs) and Confidence Intervals (CIs) for Dysregulated Glycemic Control *(HbA1c > 53 mmol/mol*) and Personality Traits				
	**Unadjusted** **OR (CI)**	**Adj. (Sex, Age)** **OR (CI)**	**Adj. (Sex, Age, and Education)** **OR (CI)**	**Adj. (Sex, Age, Education, and BMI)** **OR (CI)**	**Adj. (Sex, Age, Education, BMI, and Antihyperglycemic Agents)** **OR (CI)**
Neuroticism	0.93 (0.74–1.18)	0.93 (0.74–1.18)	0.91 (0.72–1.16)	0.91 (0.71–1.16)	0.92 (0.72–1.17)
Extroversion	1.08 (0.88–1.34)	1.08 (0.87–1.35)	1.08 (0.87–1.35)	1.05 (0.84–1.33)	1.05 (0.84–1.33)
Openness	0.96 (0.80–1.15)	0.96 (0.80–1.15)	0.97 (0.80–1.17)	0.95 (0.78–1.16)	0.95 (0.79–1.16)
Agreeableness	0.96 (0.75–1.24)	0.96 (0.74–1.23)	0.97 (0.75–1.25)	1.01 (0.77–1.32)	1.00 (0.76–1.32)
Conscientiousness	0.89 (0.70–1.14)	0.90 (0.70–1.15)	0.91 (0.71–1.17)	0.91 (0.70–1.18)	0.91 (0.70–1.17)

Reference group = well-regulated, Abb. Adj = Adjusted model.

**Table 3:** Associations between personality traits and BMI.

	Odds Ratios (ORs) and Confidence Intervals (CIs) for *BMI ≥ 25* and Personality Traits		
	**Unadjusted** **OR (CI)**	**Adj. (Sex, Age)** **OR (CI)**	**Adj. (Sex, Age, and Education)** **OR (CI)**
Neuroticism	1.33 (0.92–1.93)	1.35 (0.91–2.01)	1.34 (0.90–2.00)
Extroversion	0.70 (0.49–1.01)	0.71 (0.48–1.03)	0.71 (0.48–1.03)
Openness	0.98 (0.75–1.28)	0.96 (0.73–1.26)	0.97 (0.74–1.29)
Agreeableness	0.54 (0.34–0.86) *	0.53 (0.33–0.87) *	0.54 (0.33–0.89) *
Conscientiousness	0.74 (0.50–1.08)	0.70 (0.47–1.05)	0.71 (0.47–1.06)

Reference group = BMI < 25, Abbreviations: Adj = adjusted model, * = *p*-value < 0.05.

## Data Availability

The data are not publicly available due to privacy restrictions.
